# Introduction of a new and safe synthesis procedure for Ni-MOF-I in aqueous solution and its application for the extraction of some pesticides from different beverages†

**DOI:** 10.1039/d3ra03441k

**Published:** 2023-07-19

**Authors:** Mir Ali Farajzadeh, Nastaran Khoshnavaz, Sakha Pezhhanfar, Mohammad Reza Afshar Mogaddam

**Affiliations:** a Department of Analytical Chemistry, Faculty of Chemistry, University of Tabriz Tabriz Iran mafarajzadeh@yahoo.com mafarajzadeh@tabrizu.ac.ir +98 41 33340191 +98 41 33393084; b Engineering Faculty, Near East University Mersin 10 99138 Nicosia North Cyprus Turkey; c Food and Drug Safety Research Center, Tabriz University of Medical Sciences Tabriz Iran; d Pharmaceutical Analysis Research Center, Tabriz University of Medical Sciences Tabriz Iran

## Abstract

For the first time, this research introduces an analytical application of Ni-MOF-I, which was used as an adsorbent in a dispersive micro solid phase extraction procedure followed by dispersive liquid–liquid microextraction for the extraction and preconcentration of seven pesticides from different fruit juices. Also, Ni-MOF-I was synthesized by a new and green method with many advantages over the previously published synthesis procedures. For example, effortless and green synthesis, no need for autoclaves and ovens, and elimination of organic solvent usage are the main highlights. The synthesized Ni-MOF-I was characterized by applying nitrogen adsorption/desorption, energy-dispersive X-ray, scanning electron microscopy, Fourier transform infrared spectrophotometry, and X-ray diffraction analyses. The studied pesticides were extracted and preconcentrated by the proposed method. Then, the extracted analytes in the sedimented organic phase were injected into a gas chromatography-flame ionization detector. Acceptable analytical results such as low limits of detection (0.15–0.60 μg L^−1^) and quantification (0.50–2.0 μg L^−1^), reasonable extraction recoveries (51–80%), high enrichment factors (255–400), satisfactory relative standard deviation values of 4.8–7.2% (intra-day precision, *n* = 6) and 5.3–7.5% (inter-day precision, *n* = 4), and wide linear ranges were obtained. The proposed method can be introduced as an effective analytical technique based on Ni-MOF-I for the analysis of different pesticides in fruit beverages.

## Abstract 2

For the first time, this research introduces a new and safe synthesis process for Ni-MOF-I and its analytical application for the extraction of pesticides from juice samples.

## Graphical abstract

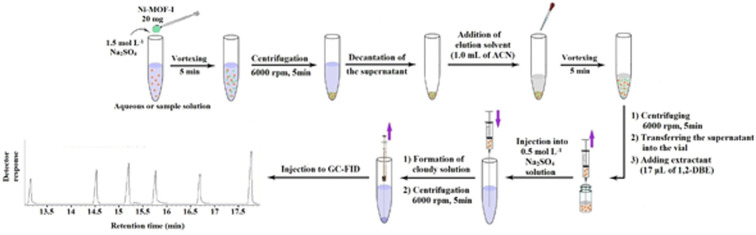

## 1. Introduction

The request for fruit-based beverages in the juice market is considerably increasing over recent years due to them containing an abundance of different phytochemicals and having valuable potential for strengthening human health.^1–3^ In addition to the mentioned positive factors, juices can be harmful for human health because of various pesticides that might be present and abundance of additive sugar.^4^ Pesticides are one of the main inputs, which are utilized to eradicate various pests in households and agriculture, heighten the output and quality of crops, decrease the energy cost, and restrict many vector-borne diseases. Despite the mentioned advantages, pesticides may put the human body at considerable danger of diseases, cause irrecoverable harm, and persist in the environment and cause the contamination of fruits, vegetables, and surface waters because of their environmental stability, capability to bioaccumulate, and toxicity.^5,6^ The residue of pesticides is out of the consumer's control.^7^ Therefore, based on the aforementioned information, it becomes imperative to develop highly sensitive, reliable, and selective analytical methods to analyze pesticide residues in foods to decrease the possible dangers for human society. These days, various types of analytical techniques such as gas chromatography (GC),^8,9^ and high performance liquid chromatography (HPLC)^10,11^ are used to determine pesticide residues in food samples including juices. Usually, these methods have great selectivity with low detection limits and high sensitivity,^12^ but most samples cannot be injected directly into them because of some limitations like the low concentration of analytes, having a complex matrix, and the intrinsic limitation of aqueous solutions in injection into GC instrument.^3^ The amounts of pesticide residues in various environments are usually under the limit of detection (LOD) of many analytical instruments, so it is necessary to integrate sample preparation steps with instrumental analysis in order to concentrate the pesticides, reduce the LODs, and acquire a reliable signal while utilizing different analytical instruments for analyzing pesticides.^13^ More than 80% of the analysis time is dedicated to the extraction, preconcentration, and preparation steps. For this reason, the critical part of most analytical procedures is sample preparation step.^14^ Methods of sample preparation should be able to isolate the matrix from the analytes^15^ and transfer them into a phase which is suitable for injection into the analysis system by extracting the targeted analytes from the sample matrix.^16^ Moreover, they should be eco-friendly, time-saving, inexpensive, and simple.^13^ The two coventional sample preparation methods which are applied for determining the pesticide residues in fruit juices are solid phase extraction (SPE) and liquid–liquid extraction (LLE).^17^ Beside the advantages they contain, they have some serious disadvantages such as high-volume use of organic solvents in the case of LLE and cartridge obstruction in the case of SPE.^18,19^ To overcome these issues, Anastassiades *et al.*^20^ introduced an alternative method over conventional SPE named dispersive solid phase extraction (DSPE) in which the adsorbent is straightly dispersed into the sample solution instead of being loaded as the cartridge. Various kinds of materials have been utilized as solid adsorbents like graphene, carbon nanotubes, metal–organic frameworks (MOFs), silica nanoparticles, mesoporous silica, *etc.*^21^ Recently, dispersive micro solid phase extraction (DμSPE) which is the miniaturized modification of DSPE has been widely developed^22^ in which it needs less than 500 mg of solid adsorbent.^4^ In comparison to DSPE, DμSPE has simpler operation, and is more economic due to the utilization of lower sorbent weights. These make the approach economical and environmentally-friendly.^22,23^ However, the main drawback of this method is the low value of enrichment factors (EFs) because of consuming mL-level of elution solvent. To solve this issue a combination of DμSPE with dispersive liquid–liquid microextraction (DLLME) is required which is an affordable and simple extraction method and results in high EFs.^24–26^ The main drawback of DLLME is the limitation of its direct application in complex matrices due to the extraction of the co-extractives that interfere on the analysis process.^27^ Considering the advantages of DμSPE and DLLME, simultaneous use of these two methods can make more benefits like the achievement of high EFs, efficient sample clean up, and low LODs and limits of quantification (LOQs).^25^ MOFs are a novel class of hybrid porous materials and a new type of three-dimensional coordination polymers formed by organic ligands and metallic clusters through coordination bonds.^28,29^ Owing to their tailorable polarity, structure flexibility, high and tunable porosity, good thermal stability, uniform cavities, adsorption capacity, and high surface area, MOFs have been used in drug delivery, photovoltaic materials, catalysis, gas purification and separation, gas storage systems, biomedicine, as adsorbents in analytical sample preparation methods, sensors in electroanalytical and spectroscopy methods, and the stationary phases of chromatographic columns.^29–31^

Many papers were reported in literature regards pesticide residues analysis in different matrices. Barrek *et al.*^32^ separated residual pesticides from olive oil by size-exclusion chromatography. After the extraction, eleven pesticides were separated and analyzed by HPLC-mass spectrometry (MS) and 20 others by GC-MS. Ma *et al.*^33^ extracted and preconcentrated four pesticides including fenpyroximate, chlorfenapyr, fipronil, and flusilazole in environmental water samples using magnetic MIL-101 in magnetic solid phase extraction procedure and analyzed by HPLC-diode array detector (DAD). Chahkandi *et al.*^34^ extracted some organophosphorus pesticides including diazinon, phosalone, fenthion, fenitrothion, and profenofos from fruit juices and water samples applying magnetic potassium substituted hydroxyapatite and analyzed by GC-flame ionization detector (FID). Michel *et al.*^35^ extracted ten fungicide, insecticide, and herbicide residues in vegetables, fruits, and cereal using LLE, SPE, and matrix solid phase dispersion. Determination was performed by reversed-HPLC-DAD. Shamsipur *et al.*^36^ used SPE coupled with DLLME and GC-MS for the extraction and determination of 20 pesticide residues from fruit juice, honey, milk, and water. Wang *et al.*^37^ developed an ultrasound-assisted DLLME method based on solidification of floating organic droplets followed by GC-FID for the extraction and determination of some pesticides including pyridaben, triazophos, es-fenvalerate, buprofezin, and λ-cyhalothrin in water samples.

This research has two main novel aspects. The first one is the introduction of a new, facile, and safe synthesis procedure for Ni-MOF-I which is done in aqueous solution. This approach is effortless, green, and needs no special synthesis instruments such as autoclaves, ovens, and toxic solvents such as *N*,*N*-dimethylformamide. The second aspect is the utilization of Ni-MOF-I for the first time in a sample preparation process for the extraction of different pesticides from juices through DμSPE-DLLME procedure and their analysis using GC-FID.

## 2. Experimental

### 2.1. Chemicals and solutions

All the target compounds (chlorpyrifos, haloxyfop-*R*-methyl, oxadiazon, diniconazole, clodinafop-propargyl, fenpropathrin, and fenoxprop-*P*-ethyl) with purity >98% were procured from Dr Ehrenstorfer (Agsburg, Germany). Sodium sulfate, potassium chloride, sodium chloride, sodium hydroxide, acetonitrile (ACN), acetone, hydrochloric acid (37%, w/w), and methanol (analytical grade) were obtained from Merck (Darmstadt, Germany). 2-Propanol was acquired from Caledon (Georgetown, Canada). Deionized water was supplied from Ghazi Co. (Tabriz, Iran). Extraction solvents including 1,1,2-trichloroethane (1,1,2-TCE), 1,1,1-trichloroethane (1,1,1-TCE), 1,2-dibromoethane (1,2-DBE), and carbon tetrachloride were purchased from Janssen (Beerse, Belgium). To synthesize the adsorbent, nickel(ii) chloride hexahydrate (NiCl_2_·6H_2_O), 1,4-benzenedicarboxylic acid (1,4-BDCA), and concentrated ammonia solution (25%, w/w) were purchased from Merck (Darmstadt, Germany). A mixture standard solution including 250 mg L^−1^ of each pesticide was provided in methanol and diluted using deionized water for daily-used solutions of interest.

### 2.2. Samples

Four commercial fruit juices including mango (the content of 200 mL juice includes sodium (13 mg), potassium (75 mg), total carbohydrates (28 g), sugar (26.25 g), vitamin A, vitamin C, calcium, iron, and natural mango puree), pineapple (the content of 200 mL juice includes sodium (22 mg), potassium (71 mg), total carbohydrates (27.5 g), sugar (27.5 g), vitamin A, vitamin C, calcium, iron, and natural pineapple puree), apple (the content of 200 mL juice includes sodium (23 mg), potassium (201 mg), total carbohydrate (26 g), vitamin A, vitamin C, calcium, iron, and natural apple puree), and peach (the contents of 200 mL juice includes sodium (22 mg), potassium (125 mg), total carbohydrate (28 g), sugars (26.25 g), vitamin A, vitamin C, calcium, iron, and natural mango puree) were purchased from a local store in Tabriz (Iran). The brand of all the commercial fruit juices used in this study Sun Ich. In addition, in order to evaluate the juices of fresh fruits, two fresh fruits including apple and orange were purchased. A juicer was used to extract the juices of these fruits. For separating the juice from its scum, centrifugation was done at 6000 rpm for 5 min. All the mentioned juices were diluted at a ratio of 1 : 1 with deionized water prior to carrying out the extraction method on them.

### 2.3. Apparatus

Chromatographic analysis and separation of the pesticides were carried out by a Shimadzu 2014 gas chromatograph (Kyoto, Japan) equipped with a split/splitless injector and an FID. A split ratio of 1 : 10 and a sampling time of 1 min were adjusted. The temperatures of the injection port and FID were set at 300 °C during the analysis. The initial temperature of the column oven was held at 60 °C for 1 min, then programmed at a rate of 18 °C min^−1^ to 300 °C, and held for 3 min to complete the separation run. Chromatographic separation was attained on a Zebron capillary column (95% dimethyl, 5% diphenyl polysiloxane) (30 m × 0.25 mm i.d, a film thickness of 0.25 μm) (Phenomenex, Torrance, CA, USA). Helium (99.999%, Gulf Cryo, Dubai, United Arab Emirates) was used as the make up and carrier gases. A flow rate of 30 mL min^−1^ and linear velocity of 30 cm s^−1^ were adjusted for the make up and carrier gases, respectively. The required hydrogen as the fuel of FID was provided by a hydrogen generator (OPGU-1500S, Shimadzu, Japan) which was set at the flow rate of 30 mL min^−1^. Additionally, the air flow rate was set to be 300 mL min^−1^. For injection of the standards and extracted samples into GC-FID, a 1.0 μL microsyringe (zero dead volume, Hamilton, Switzerland) was utilized. A Metrohm pH meter, model 654 (Herisau, Switzerland) was utilized for pH adjustment. Phase separation during the extraction process was accomplished by a Hettich centrifuge, model D-7200 (Kirchlengern, Germany). A magnetic heater-stirrer (Heidolph MR 3001 K, Germany) was applied in providing the MOF. An L46 vortex (Labinco, Breda, the Netherlands) was utilized for vortexing. The synthesized MOF's X-ray diffraction (XRD) pattern was attained by applying a Siemens D500 diffractometer (Siemens AG, Karlsruhe, Germany) at a voltage of 35 kV. It was performed at the rate and scan range of 1° min^−1^ and 4–73°, respectively. Surface characterization of the synthesized MOF was also accessed by energy-dispersive X-ray (EDX) and scanning electron microscopy (SEM) analyses utilizing a Mira 3 microscope (Tescan, Czech Republic). A Fourier transform infrared (FTIR) spectrophotometer (Bruker, Billerica, USA) was utilized in the range of 400–4000 cm^−1^ for obtaining the FTIR spectrum of the adsorbent applied in the present investigation. A BELSORP-mini II (BEL, Japan) analyzer was used to carry out nitrogen adsorption/desorption analysis for getting information about average pores diameter, pores size, and surface area of the MOF.

### 2.4. Synthesis of Ni-MOF-I

In this study, Ni-MOF-I was synthesized through a new green procedure. In comparison to the previously reported synthesis method^38^ the developed approach is more economic, time-saving, and needs no expensive and special synthesis instruments. Also, this synthesis process is environmentally friendly and not harmful for human health because unlike the previous synthesis method in which dimethylformamide was utilized as the reaction solvent, deionized water was used instead. This synthesis method entails the subsequent steps:

(a) Initially, 0.80 g of 1,4-BDCA was added as the ligand to 5.0 mL of concentrated ammonia solution and put on a stirrer. Deionized water (20 mL) was slowly added until the solution became clear. Then, the solution was poured into a burette.

(b) 1.14 g of NiCl_2_·6H_2_O was dissolved in 100 mL of deionized water in an Erlen mayer flask. Afterward, the flask was put into a water bath set at 80 °C.

(c) The solution inside the burette was added dropwisely into the flask under stirring at 300 rpm. After finishing the content of the burette, a lid was put on the flask and continued stirring for 1 h. Then, the flask was let to cool down at room temperature. The formed light green precipitate was Ni-MOF-I. Then, the content of flask was transferred into glass tubes and then were centrifuged at 6000 rpm for 5 min. Afterwards, the supernatant was decanted. The product remaining at the bottom of the tubes was vortexed with 5 mL of deionized water for 5 min, centrifuged at 6000 rpm for 5 min, and the supernatant was decanted. This process was repeated for 5 times until the product was completely washed and the decanted phase was neutralized. At the end, the obtained product was poured on a watch glass to dry at room temperature. The yield of the MOF according to the adopted synthesis method was 68.41%.

Eventually, the synthesized Ni-MOF-I was subjected to FTIR, XRD, nitrogen adsorption/desorption, EDX, and SEM analyses and after that it was applied as an adsorbent in the extraction procedure.

### 2.5. Extraction procedure

#### 2.5.1. DμSPE

Initially, into a 10 mL glass test tube, 5 mL sample solution (see Section 2.2) or deionized water spiked with 150 μg L^−1^ of each analyte was added and 1.065 g Na_2_SO_4_ was dissolved in it. Thereupon, 20 mg of Ni-MOF-I, as the adsorbent, was added into the mentioned solution. In the following, it was vortexed for 5 min to adsorb the pesticides onto Ni-MOF-I particles. Afterwards, to settle down the suspended particles of the MOF at the bottom of the tube, centrifugation was performed at the speed of 6000 rpm for 5 min. After discarding the supernatant, 1.0 mL of ACN was used to desorb the analytes from Ni-MOF-I particles. In order to provide efficient contact between ACN and the adsorbent particles for efficient elution of the analytes, vortexing was performed for 5 min. At the end, the mixture of the adsorbent particles and elution solvent was centrifuged at 6000 rpm for 5 min and the obtained supernatant was applied in the following DLLME step as a disperser solvent.

#### 2.5.2. DLLME

**Fig. 1:**
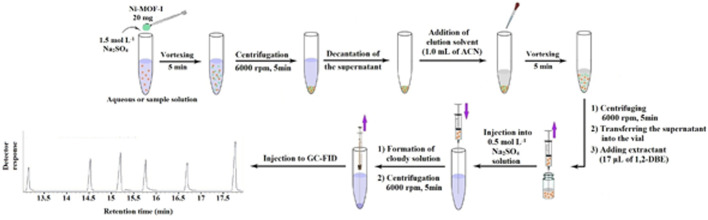
Schematic figure of the developed extraction procedure.

In a 10 mL conical bottom glass test tube, 5 mL aqueous solution of Na_2_SO_4_ (0.5 mol L^−1^) was added. Then, the ACN phase obtained from the DμSPE step, was mixed with 17 μL of 1,2-DBE (extraction solvent). Thereupon, it was quickly injected into the Na_2_SO_4_ solution by using a 5 mL glass syringe. The formation of the cloudy state indicated that DLLME was done correctly. Then, the cloudy solution was centrifuged at 6000 rpm for 5 min. Eventually, 1 μL of the sedimented organic phase at the bottom of the test tube was injected into the GC-FID. Fig. 1 demonstrates the overall schematic of the applied extraction and preconcentration procedure.

### 2.6. Calculation of EF and extraction recovery (ER)

ER and EF were utilized to appraise efficiency of the extraction in various experimental conditions. The equations used to calculate their values are written below.1
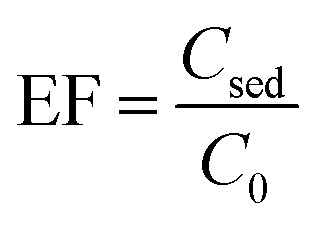
2
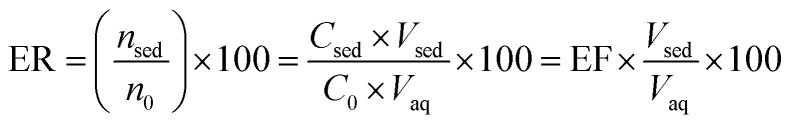


The ratio of pesticide concentration in the extraction solvent (*C*_sed_) to the pesticide concentration in the initial sample solution (*C*_0_) is described as EF. The ER is the ratio of the extracted pesticide amount (*n*_sed_) to its initial amount (*n*_o_) multiplied by 100. Moreover, the term *V*_sed_ indicates volume of the settled organic phase in DLLME and *V*_aq_ shows the initial aqueous sample volume.

## 3. Results and discussion

### 3.1. Characterization of Ni-MOF-I

To identify the synthesized MOF, some characterization analyses were performed such as FTIR, XRD, nitrogen adsorption/desorption, EDX, and SEM. By comparing the obtained results with the results reported previously,^38,39^ it was concluded that the synthesized adsorbent is Ni-MOF-I.

**Fig. 2:**
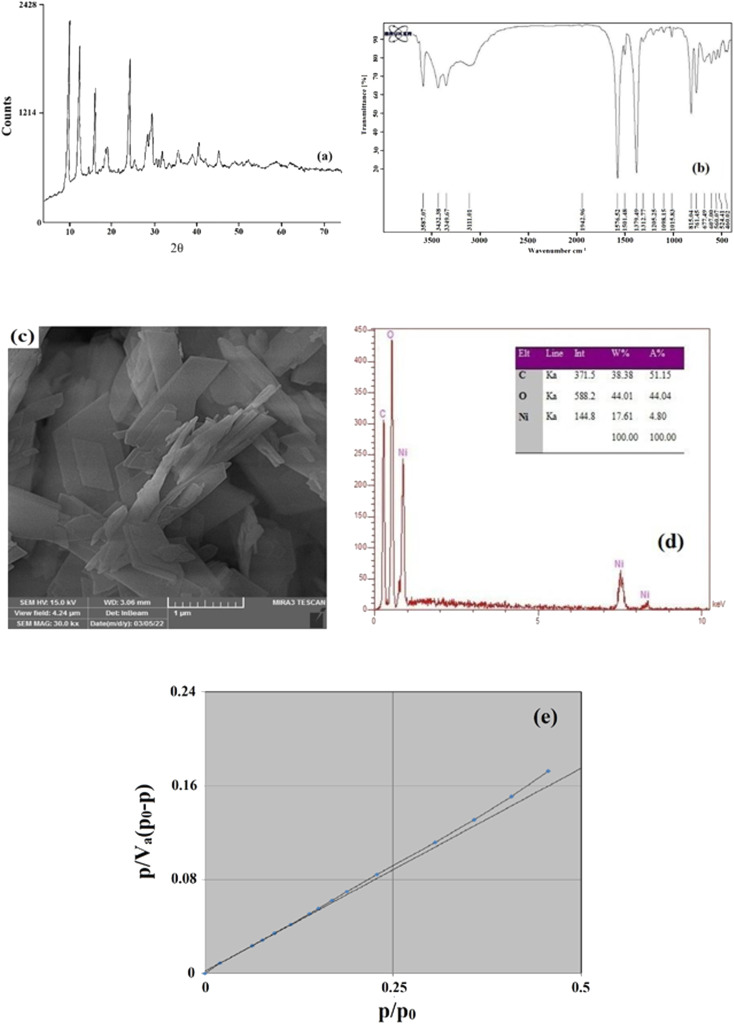
XRD pattern (a), FTIR spectrum (b), SEM image (c), EDX data (d), and BET plot (e) of Ni-MOF-I.

XRD is an influential and essential analysis for diagnosing and validating the creation of the required crystalline compound. For this purpose, XRD analysis was done in the range of 4–73°. The outcome XRD pattern of Ni-MOF-I is shown in Fig. 2a. Same peaks are observed at 2*θ* values of around 10, 12, 16, 18, 19, 24, 28, and 29°. Based on the attained XRD pattern, some acute and distinct peaks are observed which is the reason for the crystalline structure and successful formation of Ni-MOF-I. Furthermore, the perfect match between the obtained pattern and the previously reported pattern^32^ verifies the successful synthesis of Ni-MOF-I in this research.

FTIR spectrometry was also applied to identify the formation of the intended MOF. Fig. 2b shows the FTIR spectrum of the synthesized Ni-MOF-I. Two absorption peaks at 1576.52 and 1379.49 cm^−1^ are related to the symmetric and asymmetric stretching vibration of the –COO– functional group of the ligand coordinated to the metallic center. The presence of peaks at 460.02, 607.00, and 677.49 cm^−1^ are ascribed to the formation of the metal–oxygen bond between the carboxylic group of 1,4-BDCA and Ni (Ni–O). The peaks at 1501.48 and 3432.38 cm^−1^ are attributed to the stretching vibration of CH and OH, respectively. Also, the peaks at 761.05 and 815.05 cm^−1^ are related to the C–H and C=C bonds present in the ligand section of the synthesized MOF. According to the above-mentioned results and their perfect agreement with the previously published FTIR spectrum of the desired MOF,^33^ it reveals that Ni-MOF-I synthesis was done correctly.

The morphology of the surface of the synthesized MOF can be investigated by SEM. So, high resolution image of the MOF which is shown in Fig. 2c, was obtained to attain the surface morphology of Ni-MOF-I. As can be observed, the Ni-MOF-I shows a layer-cuboid morphology with plain surface which provides an appropriate adsorption surface for the investigated pesticides.

EDX analysis was applied to reveal the presence of elements in the structure of the synthesized MOF and also to obtain information about the MOF's purity. The results are shown in Fig. 2d. As can be clearly noticed, there are some main and sharp peaks corresponding to C, O, and Ni which confirm the presence of composing elements in Ni-MOF-I. No appearance of peaks related to other elements is the proof of the purity of the synthesized MOF. The EDX analysis reveals that the surface of Ni-MOF-I contains 17.61% nickel, 44.01% oxygen, and 38.38% carbon.

To get information regarding the surface area, average pores diameter, and total pores volume of Ni-MOF-I, nitrogen adsorption/desorption analysis was applied (Fig. 2e). The results of the analysis were reported as follows: 0.055 cm^3^ g^−1^ for total pores volume, 17.641 nm for average pores diameter, and 12.503 m^2^ g^−1^ for the surface area of the MOF. The mentioned data were obtained from the BET data.

### 3.2. Optimization of parameters in DμSPE

#### 3.2.1. Evaluation of Ni-MOF-I weight

**Fig. 3:**
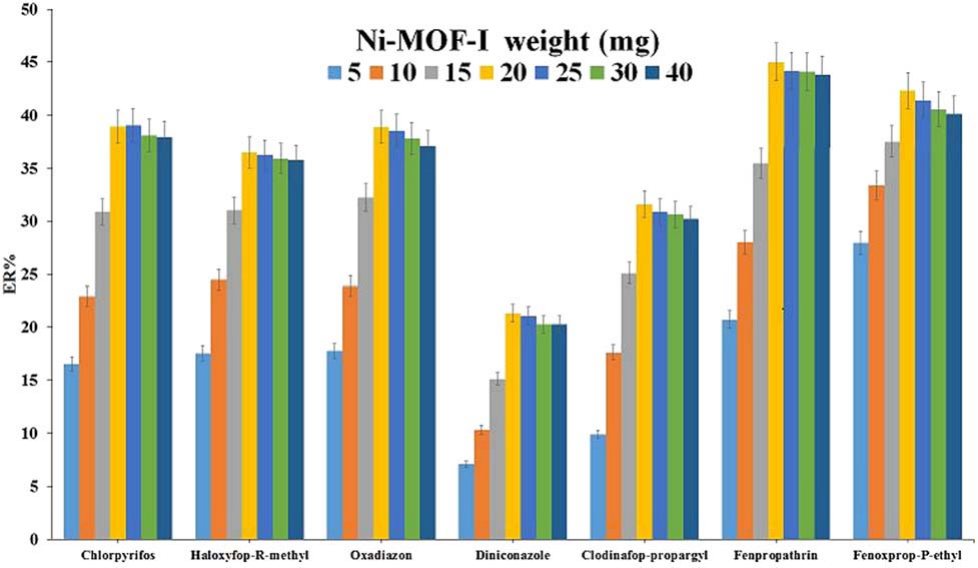
Evaluation of Ni-MOF-I weight. Extraction conditions: DμSPE procedure: aqueous solution, 5 mL deionized water containing 1.0 mol L^−1^ Na_2_SO_4_ spiked with 150 μg L^−1^ of each analyte without pH adjustment; vortex time in adsorption step, 5 min; desorption solvent (volume), ACN (1.0 mL); vortex time in desorption step, 5 min; and centrifugation speed and time, 6000 rpm and 5 min, respectively. DLLME procedure: aqueous phase, 5 mL deionized water without pH adjustment and salt addition; extraction solvent (volume), 1,2-DBE (31 μL); centrifugation rate, 6000 rpm; and centrifugation time, 5 min. The error bars show the minimum and maximum of three repeated determinations.

The adsorbent weight in adsorbent-based methods has a significant effect on the extraction efficiency because the amount of the adsorbent has a direct effect on the amount of the adsorbed analytes. For evaluating this parameter, various amounts of Ni-MOF-I (5, 10, 15, 20, 25, 30, and 40 mg) were investigated. As illustrated in Fig. 3, 20 mg of the MOF indicates the most efficient performance compared to the tested weights in this research. Reducing the adsorbent weight from 20 mg decreases ER values because of the lack of adequate surface area to adsorb the analytes. Also, the little reduction of ERs in the amounts above 20 mg of the adsorbent occurs due to the adsorbent particles agglomeration and not being dispersed efficiently into the aqueous phase. Accordingly, the weight of 20 mg was considered as the optimum amount of the adsorbent for the rest of the optimization steps.

#### 3.2.2. Study of ionic strength

**Fig. 4:**
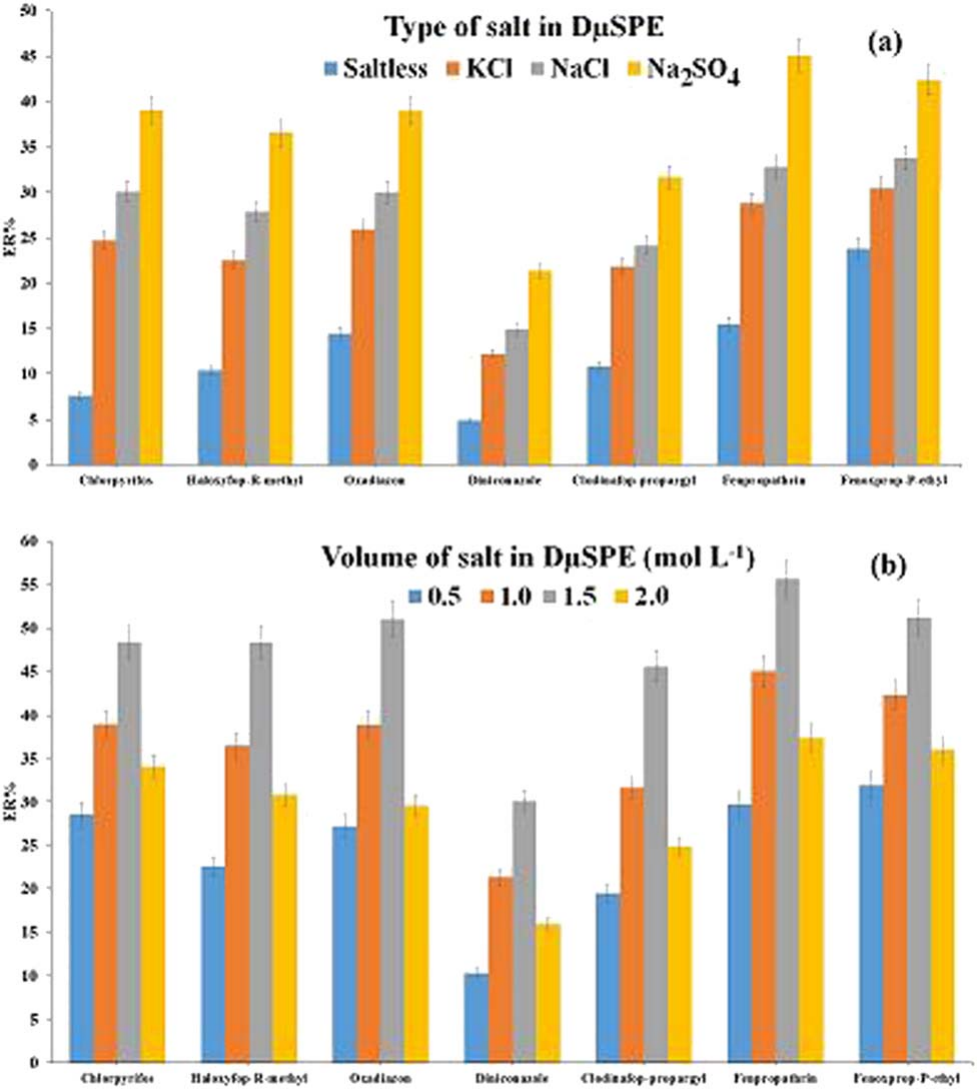
Study of ionic strength in DμSPE. (a) Salt type and (b) concentration of Na_2_SO_4_. Extraction conditions: are the same as those used in Fig. 3, except 20 mg of Ni-MOF-I was used.

The aqueous phase ionic strength in the DμSPE step is one of the effective factors which should be optimized. Salt addition enhances the aqueous phase ionic strength and it may enhance the method ER values by reducing the analytes solubility in the aqueous solution which leads to enhancement of the analytes partition onto the sorbent surface which is named salting-out effect. Also, salt addition can have an adverse effect on the extraction procedure in which the aqueous phase viscosity enhances by salt addition, which avoids the analytes migration from the aqueous phase onto the adsorbent and decreases ERs. This effect is named salting-in effect. To evaluate the effect of this parameter in the present research, three different solutions each containing 1.0 mol L^−1^ of KCl, NaCl, and Na_2_SO_4_, separately were prepared and finally the results were compared with the results of saltless solution. As shown in Fig. 4a, in Na_2_SO_4_ solution the highest extraction efficiency is obtained compared to the other solutions. So, Na_2_SO_4_ was chosen as the salting-out agent and then in order to appraise the impact of Na_2_SO_4_ concentration, several concentrations of Na_2_SO_4_ containing 0.5, 1.0, 1.5, and 2.0 mol L^−1^ were studied. The illustrated conclusions in Fig. 4b show that ER values are heighten with increasing concentration of Na_2_SO_4_ from 0.5 to 1.5 mol L^−1^ and then reduced by increasing Na_2_SO_4_ concentration over 1.5 mol L^−1^. Because the concentrations less than 1.5 mol L^−1^ are insufficient for salting-out of the pesticides, the efficiency is low and at the higher salt concentration (2.0 mol L^−1^) due to salting-in effect, ERs are reduced. As a result, 1.5 mol L^−1^ Na_2_SO_4_ was chosen as the optimum concentration in the further steps.

#### 3.2.3. Optimization of solution pH

Because the pesticides and adsorbent stability may be varied at different pH values, variation of the solution pH in DμSPE can be a noteworthy and effective parameter on the resulted ER values. So, it is vital to evaluate the impact of the solution pH on the extraction efficiency. To appraise this factor, the solution pH was adjusted in the range of 3–11 (at 2-unit intervals) by adding 1 mol L^−1^ of NaOH or HCl solution. Fig. S1† shows that the highest ER values are attained in neutral pH. The diminution of ERs in alkaline or acidic pHs can be because of: (1) the instability of the adsorbent at highly acidic or alkaline pHs, and (2) the decomposition of the analytes at the mentioned pHs. Since the samples pH utilized in this research was about 7 after dilution with deionized water, the further steps were conducted without pH variation.

#### 3.2.4. Study of adsorption time

Appropriate adsorbent dispersion into the aqueous solution containing the analytes increases the collisions between pesticides and MOF particles which leads to heighten the pesticides adsorption onto the adsorbent particles. Therefore, studying the effect of vortexing time of the adsorption stage is critical. In this research, to achieve the optimum value, different vortexing times (1.0, 3.0, 5.0, 7.0, 9.0, and 11.0 min) were investigated. According to the presented results in Fig. S2,† 5 min vortexing has the highest ER values and was considered as the optimum vortexing time for the further steps.

#### 3.2.5. Optimization of the type and volume of elution/disperser solvent

**Fig. 5:**
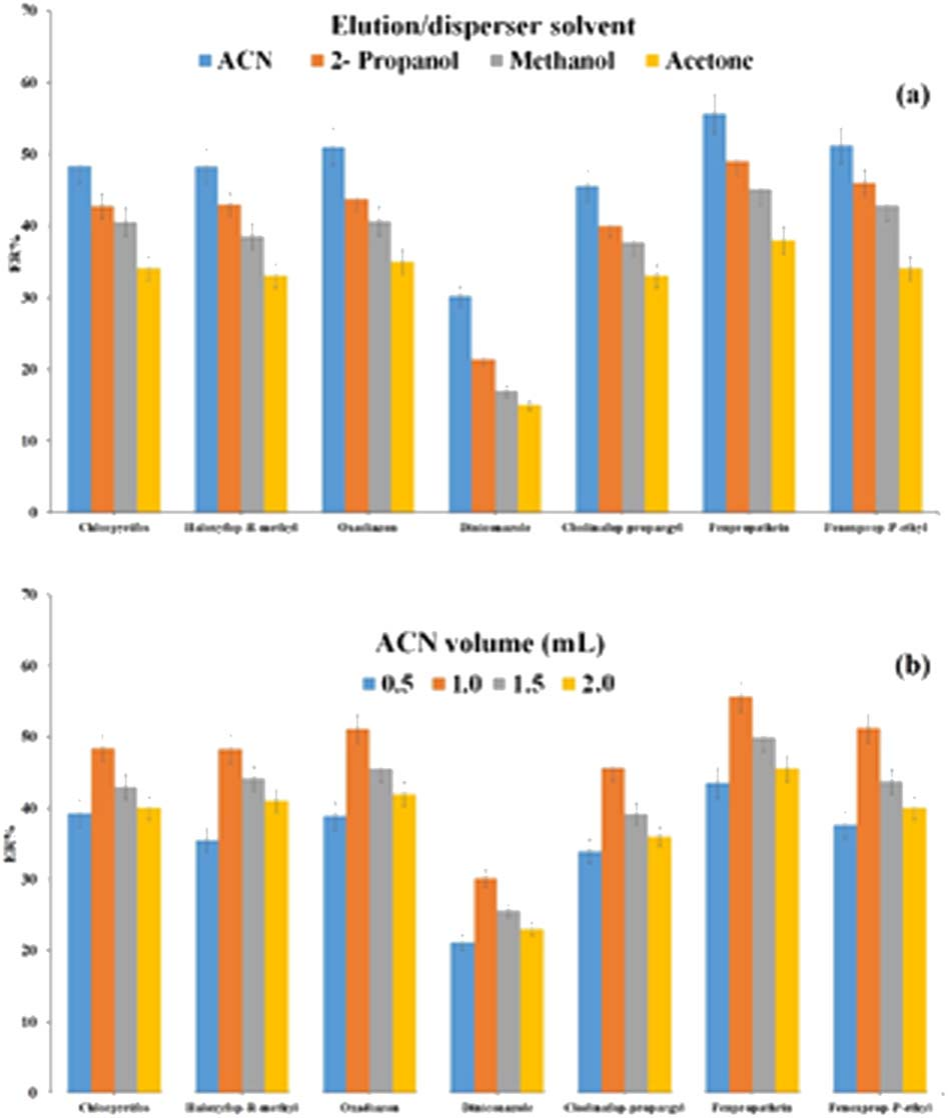
Optimization of type (a) and volume (b) of elution/disperser solvent. Extraction conditions: are the same as those used in Fig. 4, except 1.5 mol L^−1^ Na_2_SO_4_ was used.

In this research, elution solvent is applied for the analytes desorption in DμSPE and also this is one component of DμSPE which is in common with DLLME because it is used as a disperser solvent in DLLME. So, according to aforementioned facts, this solvent has considerable effects on both steps and this fact reveals the necessity of choosing a proper elution/disperser solvent. An appropriate elution/disperser solvent is a solvent that is miscible in both organic and aqueous phases. Also, it has to be able to desorb the analytes successfully from the surface of Ni-MOF-I. For this intention, 1.0 mL of different solvents which possess these traits like ACN, 2-propanol, methanol, and acetone were examined. As shown in Fig. 5a, the best ERs are attained by employing ACN compared to the other used solvents. So, ACN was chosen as optimum elution/disperser solvent for the upcoming steps. Afterwards, to assess the impact of ACN volume, several volumes of this solvent (0.5, 1.0, 1.5, and 2.0 mL) were tested. The final results in Fig. 5b illustrate that 1.0 mL of ACN has the highest extraction efficiency compared to the other volumes. The reason for the reduction of ERs in the lower volume (0.5 mL) of ACN is based on two facts: (1) inability to desorb the adsorbed analytes from the adsorbent surface, and (2) deficient formation of the cloudy state in DLLME stage. Also, the volumes over 1.0 mL of ACN in the DLLME step cause diminution of the aqueous solution polarity and enhancement of the analytes solubility in the aqueous phase. So, the transfer of the analytes into the extraction solvent is reduced. Finally 1.0 mL ACN was selected as the elution/disperser solvent for the further tests.

#### 3.2.6. Optimization of desorption time

Vortexing was employed for desorbing the pesticides from the surface of the applied MOF in the desorption step. Several vortexing times including 1.0, 3.0, 5.0, 7.0, 9.0, and 11.0 min were tested to identify the time with the highest desorption efficiency. The obtained data in Fig. S3† confirm that 5 min vortexing is sufficient to desorb the pesticides from the adsorbent surface and it was chosen as the optimum vortexing time for the further investigations.

### 3.3. Optimization of parameters in DLLME

#### 3.3.1. Study of extraction solvent type and volume

**Fig. 6:**
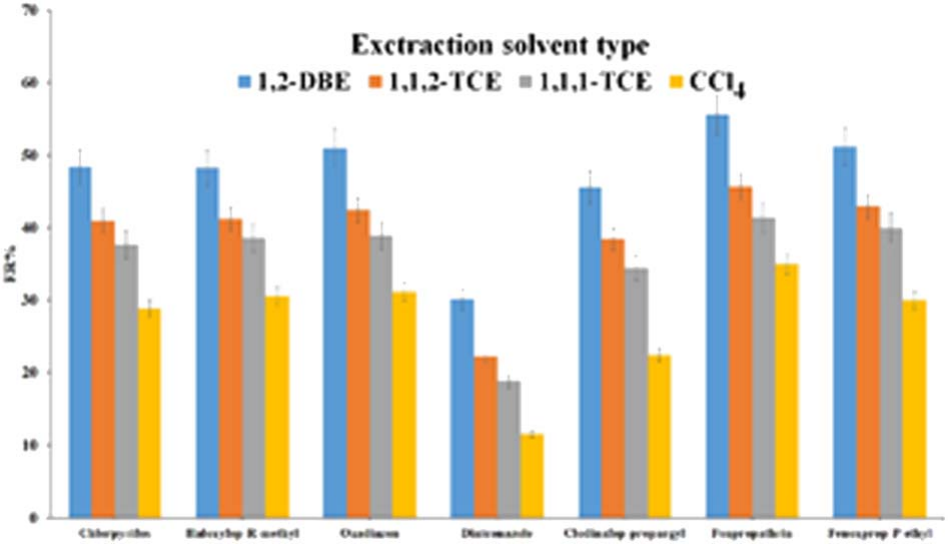
Study of extraction solvent type. Extraction conditions: are the same as those used in Fig. 5, except 1.0 mL ACN was used as the elution solvent.

Choosing the best extraction solvent is very important and vital in DLLME. This solvent should have several properties including ability to extract the pesticides as much as possible, being water-immiscible and miscible with the elution/disperser solvent, demonstrating favorable chromatographic behavior, capability of forming a stable cloudy state in the presence of ACN, and to be sedimented at the bottom of the test tube after centrifugation due to its higher density than water. The solvents such as 1,2-DBE, carbon tetrachloride, 1,1,1-TCE, and 1,1,2-TCE are the extraction solvents which have the aforementioned properties and were used for optimization in this experiment. In order to attain a constant sedimented phase volume (10 ± 0.5 μL) in each experiment, the volumes of 27, 22, 28, and 25 of 1,2-DBE, carbon tetrachloride, 1,1,2-TCE, and 1,1,1-TCE were utilized, respectively. According to the results shown in Fig. 6 1,2-DBE has the highest extraction efficiency and therefore was opted as the optimal extraction solvent. Extraction solvent volume is an effective parameter in DLLME because this parameter directly affects the sedimented organic phase volume, LODs, EFs, and ERs of the analytes. To evaluate this parameter impact, different volumes of 1,2-DBE including 26, 31, 36, and 41 μL were examined. It is worth mentioning that when the volume of 1,2-DBE increased, ERs enhanced and EF values decreased due to dilution effect. By heightening the extraction solvent volume, the sedimented organic phase volume enhanced and dilution of the analytes occurred into it. Also, in volumes less than 26 μL, the sedimented organic phase volume was less than 10 μL which was too low to collect and handle and led to a decrement in the method repeatability. Based on the obtained data in Fig. S4,† the optimum volume of the extraction solvent was 26 μL.

#### 3.3.2. Optimization of aqueous solution pH

To evaluate the impact of this parameter on the extraction efficiency in DLLME stage, the pH of the aqueous solution was adjusted at 3.0, 5.0, 7.0, 9.0, and 11.0 by using 1 mol L^−1^ of NaOH or HCl solution. By analyzing the obtained results in Fig. S5,† it becomes vivid that the solution with neutral pH has the highest extraction efficiency. This results can stem from the fact that strict acidic or basic pHs play a significant role in decomposition of the analysts. So, in the following, deionized water without pH adjustment was used as the aqueous phase in DLLME step.

#### 3.3.3. Impact of ionic strength

As mentioned before, aqueous solution ionic strength is an effectual factor on the ERs of the analytes. To investigate this issue, different solutions containing different salts including KCl, NaCl, and Na_2_SO_4_ (0.50 mol L^−1^ of each) were used as the aqueous phase in DLLME step and the final outcomes were compared to the saltless solution. The experimental results in Fig. S6(a)† illustrates that Na_2_SO_4_ had the highest effect in enhancement of the extraction efficiency. So, the further experiments were conducted by applying Na_2_SO_4_ as the optimum salt. After opting the type of salt, the impact of salt concentration should be evaluated. For this purpose, several concentrations of Na_2_SO_4_ including 0.50, 1.0, 1.5, and 2.0 mol L^−1^ were appraised. Comparing the obtained data (Fig. S6(b)†) shows that 0.50 mol L^−1^ of Na_2_SO_4_ resulted in better ERs. At the concentrations above 0.50 mol L^−1^, due to the increase in the solution viscosity and preventing the migration of pesticides from the aqueous phase into the extraction solvent, the ERs dwindled. Hence, 0.50 mol L^−1^ was chosen as the optimum salt concentration.

### 3.4. The reusability of Ni-MOF-I

One of the effective factors in evaluating the efficiency of an adsorbent is its reusability in the repetitive extraction processes. In order to study this factor, the MOF was subjected to repetitive extraction cycles. Before repeating the extraction, the used adsorbent was eluted twice (each time by 0.5 mL of ACN) and vortexing for 5 min to prevent carry-over. Based on the obtained data, the adsorbent showed proper adsorption ability of the analytes after three cycles without significant variations in the ERs. The results showed that Ni-MOF-I is a sufficient adsorbent for utilization in repetitive extraction cycles.

### 3.5. The interaction mechanisms among the pesticides and Ni-MOF-I

The adsorption of the pesticides on the MOF with appreciable ERs is due to the adsorptive interactions among the pesticides and Ni-MOF-I. By considering the chemical structures of Ni-MOF-I and studied pesticides, it can be concluded that a noncovalent interaction called π–π stacking happens among the MOF's ligand and the cyclic sections of fenoxaprop-*P*-ethyl, fenpropathrin, clodinafop propargyl, oxadiazon, haloxyfop-*R*-methyl, and chlorpyrifos. Halogen bond is another significant intermolecular interaction which is based on the noncovalent interaction among a Lewis base, π-system, or an anion and the halogen atom in another compound. In this research, halogen bonds emerge between the oxygen atoms of Ni-MOF-I and chlorine in chlorpyrifos, oxadiazon, and fenoxaprop-*P*-ethyl, and also fluoride and chlorine in clodinafop propargyl and haloxyfop-*R*-methyl. Based on the descriptions mentioned above, efficient adsorptive intermolecular bonds are formed among Ni-MOF-I and the studied pesticides.

### 3.6. Method validation

A sign of the success of a new method is its obtained analytical figures of merit such as LOQ, LOD, EF, ER, relative standard deviation (RSD), linear range (LR), and coefficient of determination (*r*^2^). The abstract of the above-mentioned quantitative parameters is given in Table 1. The EFs and ERs of this approach were in the ranges of 255–400 and 51–80%, respectively. The RSD values were computed at the concentration of 50 μg L^−1^ of each pesticide. The RSDs for intra- (*n* = 6) and inter-day (*n* = 4) precisions were in the ranges of 4.8–7.2 and 5.3–7.5%, respectively. The LOQ and LOD calculations were based on signal-to-noise ratios of 10 and 3, respectively. The LOQ and LOD values of this method were in the ranges of 0.50–2.0 and 0.15–0.60 μg L^−1^, respectively. Calibration curves were plotted at different concentrations of pesticides utilizing standard solutions. The *r*^2^ values for this method were greater than or equal to 0.995 which indicate the good linearity of the method. The LRs were in the range of 2.0–500 μg L^−1^. Low LOD, LOQ, and RSD values, wide LRs, and applying little amounts of organic solvents and Ni-MOF-I are the main privileges of this current procedure.

**Table 1:** The obtained figures of merit for the proposed analytical method

Analyte	LOD^a^	LOQ^b^	LR^c^	*r* ^2^ d	RSD^e^ (%)		EF ± SD^f^	ER ± SD^g^
					Intra-day	Inter-day		
Chlorpyrifos	0.60	2.0	2.0–500	0.996	5.6	6.0	375 ± 25	75 ± 5
Haloxyfop-*R*-methyl	0.30	1.0	1.0–500	0.996	5.1	5.9	380 ± 15	76 ± 3
Oxadiazon	0.30	1.0	1.0–500	0.995	4.8	5.3	390 ± 15	78 ± 3
Clodinafop-propargyl	0.15	0.50	0.50–500	0.998	7.2	7.5	255 ± 20	51 ± 4
Clodinafop propargyl	0.25	0.85	0.85–500	0.997	6.2	7.2	350 ± 10	70 ± 2
Fenpropathrin	0.25	0.85	0.85–500	0.995	5.9	7.1	400 ± 15	80 ± 3
Fenoxaprop-*P*-ethyl	0.30	1.0	1.0–500	0.997	5.0	5.8	360 ± 20	72 ± 4

a Limit of detection (S/N = 3) (μg L^−1^).

b Limit of quantification (S/N = 10) (μg L^−1^).

c Linear range (μg L^−1^).

d Coefficient of determination.

e Relative standard deviation at a concentration of 50 μg L^−1^ of each analyte for intra- (*n* = 6) and inter-day (*n* = 4) precisions.

f Enrichment factor ± standard deviation (*n* = 3).

g Extraction recovery ± standard deviation (*n* = 3).

### 3.7. Analysis of real samples

**Fig. 7:**
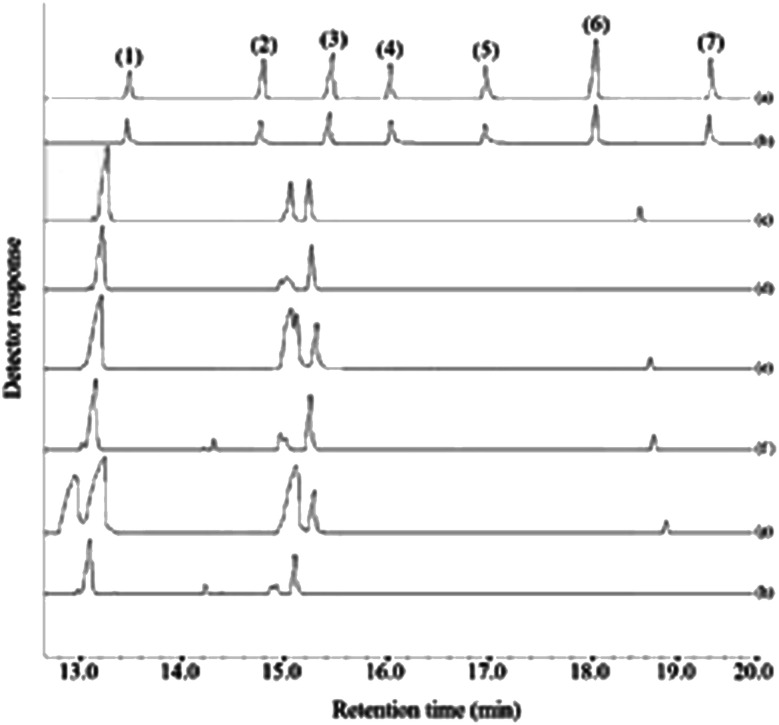
Typical GC-FID chromatograms of: (a) standard solution (250 mg L^−1^ of each pesticide in methanol), (b) aqueous standard solution at a concentration of 150 μg L^−1^ of each pesticide, (c) pineapple juice, (d) fresh orange juice, (e) peach juice, (f) fresh apple juice, (g) mango juice, and (h) commercial apple juice. In all cases, except chromatogram (a) the proposed method was performed and 1 μL of the sedimented phase was injected into the separation system. Peaks identification: (1) chlorpyrifos, (2) haloxyfop-*R*-methyl, (3) oxadiazon, (4) clodinafop propargyl, (5) fenpropathrin, and (6) fenoxaprop-*P*-ethyl.

In this research, the applicability of the method was evaluated by analyzing four commercial fruit juices including mango, pineapple, apple, and peach juices and two fresh orange and apple juices. For this purpose, deionized water and the fruit juices were spiked at 30 and 60 μg L^−1^ of each pesticides, extracted in the optimum conditions, and eventually the obtained sedimented phase injected into GC-FID. The achieved relative recoveries of the samples compared with deionized water are summarized in Table 2. As it can be deduced from the obtained results, the analyzed juices matrices have no significant effect on the present method. Therefore, this method can be used as an efficient and appropriate method for the extracting and analyzing the studied pesticides in the aforementioned samples. Fig. 7 indicates the GC-FID chromatograms of a standard solution (250 mg L^−1^ of each pesticide) which was directly injected, the extracted standard aqueous solution (containing 150 μg L^−1^ of each pesticide), and the extracted unspiked juices under the optimum conditions. According to the chromatograms, it can be seen that the chromatograms of the real samples do not show traces of the presence of any of the surveyed pesticides.

**Table 2:** Study of matrix effect in the juice samples spiked at different concentrations. All samples were diluted with deionized water at a ratio of 1 : 1

Analytes	Mean relative recovery ± standard deviation (*n* = 3)					
	Fresh apple juice	Peach juice	Pineapple juice	Apple juice	Mango juice	Fresh orange juice
**All samples were spiked with each analyte at a concentration of 30 μg L^−1^**						
Chlorpyrifos	95 ± 2	97 ± 2	94 ± 3	97 ± 2	92 ± 2	96 ± 2
Haloxyfop-*R*-methyl	97 ± 2	93 ± 3	96 ± 3	92 ± 2	91 ± 2	91 ± 2
Oxadiazon	96 ± 3	90 ± 3	90 ± 3	90 ± 4	93 ± 4	92 ± 2
Diniconazole	91 ± 2	92 ± 4	91 ± 3	91 ± 3	90 ± 3	97 ± 2
Clodinafop-propargyl	94 ± 3	88 ± 4	93 ± 2	92 ± 2	95 ± 2	96 ± 2
Fenpropathrin	93 ± 2	98 ± 4	94 ± 3	93 ± 3	92 ± 4	93 ± 2
Fenaxaprop-*P*-ethyl	90 ± 4	94 ± 3	92 ± 4	95 ± 3	89 ± 3	90 ± 3

**All samples were spiked with each analyte at a concentration of 60 μg L^−1^**						
Chlorpyrifos	95 ± 3	93 ± 2	94 ± 2	97 ± 3	92 ± 3	96 ± 2
Haloxyfop-*R*-methyl	90 ± 4	90 ± 3	91 ± 2	92 ± 2	89 ± 2	91 ± 3
Oxadiazon	91 ± 2	96 ± 2	92 ± 2	87 ± 3	93 ± 2	89 ± 3
Diniconazole	93 ± 3	92 ± 3	96 ± 2	95 ± 2	85 ± 3	98 ± 3
Clodinafop-propargyl	92 ± 3	87 ± 4	89 ± 2	89 ± 3	94 ± 4	99 ± 2
Fenpropathrin	94 ± 4	98 ± 3	93 ± 2	93 ± 3	92 ± 4	94 ± 2
Fenaxaprop-*P*-ethyl	97 ± 3	89 ± 2	95 ± 2	91 ± 2	90 ± 3	97 ± 3

### 3.8. Method comparison

Table 3 contains comparison of the analytical characteristics (EF, ER, LOD, RSD, and LR) of the proposed method in this research and other previously reported methods for analyzing the mentioned pesticides. According to this table, the present method has comparable LRs with most of the other methods. Also, the RSDs are comparable with or lower than those attained for the mentioned methods. Moreover, the EFs and ERs of the studied approach are comparable with those of the other mentioned techniques. In addition, the proposed method LOQs and LODs are lower than or comparable with the reported methods data, except for one method in which mass spectrometry has been used as the detector instead of FID which is intrinsically more sensitive than FID. Eventually, it can be said that this method is an effective and efficient analytical approach due to high EFs, reasonable ERs, low LODs and LOQs, applying little amount of Ni-MOF-I as the adsorbent, and green synthetic procedure of the MOF.

**Table 3:** Comparison of the introduced method with some other methods used for analysis of the studied pesticides

Method	Sample	LOD^a^	LOQ^b^	LR^c^	*r* ^2^ d	RSD^e^ (%)	EF^f^	ER^g^ (%)	Ref.
DSPE-DLLME-GC-FID^h^	Fruit juices	0.32–0.76	1.10–2.60	1.1–2000	0.994–0.999	4.6–8.4	680–840	68–84	40
HF-LPME-GC-MS^i^	Water	0.3–0.8	1.0	1–5000	0.997–0.999	6–9	134–240	—	41
SBSE-SFOD-GC-MS^j^	Tomato juice	0.007–0.014	0.023–0.047	0.037–2000	0.998	6–9	—	76–90	42
HS-SPME-GC-MS^k^	Wine	0.1	0.4	0.5–150	0.995	13.5	—	—	43
DμSPE-DLLME-GC-FID^l^	Fruit beverages	0.15–0.60	0.50–2.0	2.0–500	0.995–0.997	4.8–7.2	255–400	51–80	Present method

a Limit of detection (μg L^−1^).

b Limit of quantification (μg L^−1^).

c Linear range (μg L^−1^).

d Coefficient of determination.

e Relative standard deviation.

f Enrichment factor.

g Extraction recovery.

h Dispersive solid phase extraction-dispersive liquid–liquid microextraction-gas chromatography-flame ionization detection.

i Hollow fiber-liquid phase microextraction-gas chromatography-mass spectrometry.

j Stir bar sorptive extraction-solidification of floating organic droplet-gas chromatography-mass spectrometry.

k Headspace-solid phase microextraction-gas chromatography-mass spectrometry.

l Dispersive micro solid phase extraction-dispersive liquid–liquid microextraction-gas chromatography-flame ionization detection.

## 4. Conclusions

For the first time in this research, Ni-MOF-1 was synthesized through a new, facile, and safe process which was done in deionized water. The approach was green, economic, and time-saving. The MOF was also applied as an efficient adsorbent in DμSPE-DLLME-GC-FID method for the extraction, preconcentration, and determination of some pesticides in fruit juice samples. Furthermore, characterization of the synthesized MOF was done by applying nitrogen adsorption/desorption, SEM, EDX, FTIR, and XRD techniques. The proposed analytical method showed satisfactory analytical figures of merit such as low LODs (0.15–0.60 μg L^−1^), and LOQs (0.50–2.0 μg L^−1^), high EFs (255–400), acceptable ERs (51–80%), good repeatability (RSD ≤ 7.2%), and wide LRs (2.0–500 μg L^−1^). Green synthesis of the sorbent and applying low weight of MOF during the analytical approach, were the other important advantages of the proposed procedure. Also, no critical matrix effect was detected in the real samples analysis. Based on the obtained results, it can be concluded that this method can be utilized in the extraction, preconcentration, and analyzing low concentrations of the studied pesticides in different fruit juices with high reliability.

## Author contributions

Mir Ali Farajzadeh: analytical methodology, synthesis methodology, and editing the manuscript. Nastaran Khoshnavaz: analytical methodology, synthesis methodology, MOF synthesis, analytical analysis, and writing the manuscript. Sakha Pezhhanfar: analytical methodology, synthesis methodology, MOF synthesis, and editing the manuscript. Mohammad Reza Afshar Mogaddam: analytical methodology.

## Conflicts of interest

The authors declare that they have no competing interests.

## Supplementary Material

RA-013-D3RA03441K-s001
