# Supplementary material for: Introduction of a new and safe synthesis procedure for Ni-MOF-I in aqueous solution and its application for the extraction of some pesticides from different beverages
Source: RSC Adv. 2023 Jul 19;13(31):21673–84. doi: 10.1039/d3ra03441k (PMC10354589; doi:10.1039/d3ra03441k)
Supplement: RA-013-D3RA03441K-s001 [file RA-013-D3RA03441K-s001.pdf]

Electronic Supplementary Material (ESI) for RSC Advances.
This journal is © The Royal Society of Chemistry 2023

Supplementary materials

Introduction of a new and safe synthesis procedure for Ni-MOF-I in aqueous solution and its application for the extraction of some pesticides from different beverages

Mir Ali Farajzadeh *, [a, b], Nastaran Khoshnavaz [a], Sakha Pezhhanfar [a], Mohammad Reza Afshar Mogaddam [c, d]

[a] Department of Analytical Chemistry, Faculty of Chemistry, University of Tabriz, Tabriz, Iran

[b] Engineering Faculty, Near East University, 99138 Nicosia, North Cyprus, Mersin 10, Turkey

[c] Food and Drug Safety Research Center, Tabriz University of Medical Sciences, Tabriz, Iran

[e] Pharmaceutical Analysis Research Center, Tabriz University of Medical Sciences, Tabriz, Iran

*Corresponding author: M.A. Farajzadeh

Tel.: +98 41 33393084

Fax: +98 41 33340191

E-mail address: mafarajzadeh@yahoo.com; mafarajzadeh@tabrizu.ac.ir

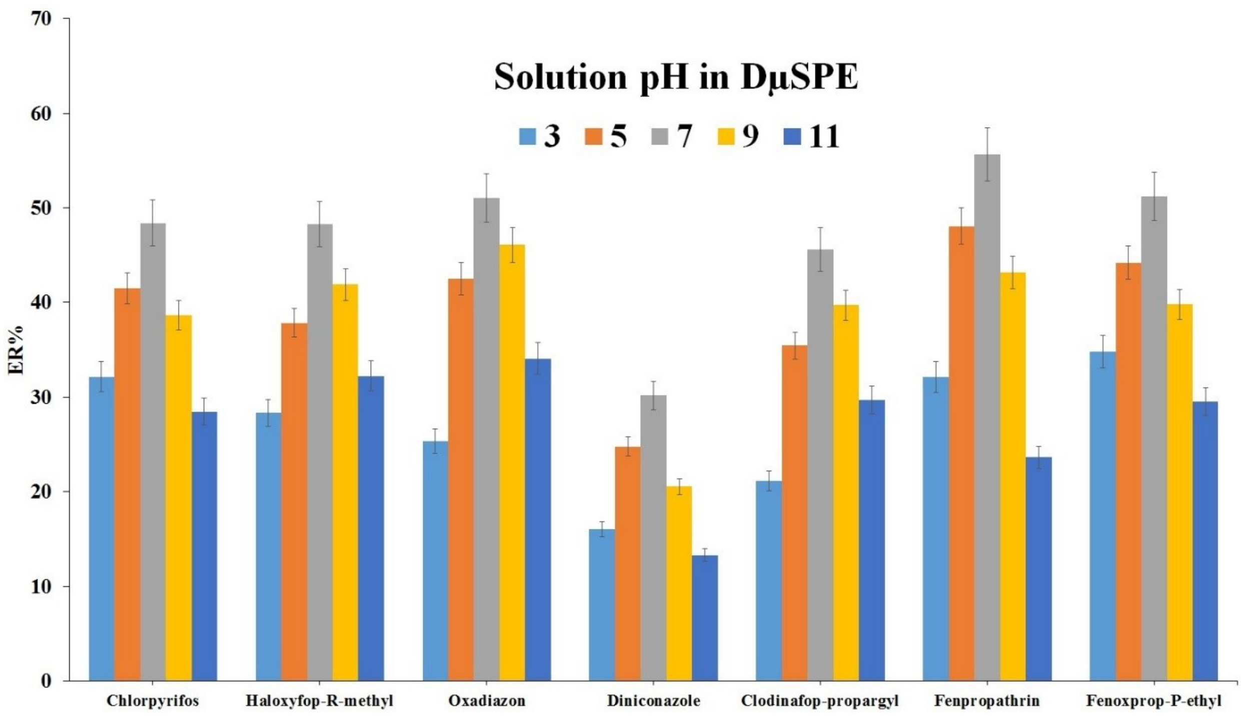

Fig. S1. Study of pH in DμSPE.

Extraction conditions: DμSPE procedure: aqueous solution, 5 mL deionized water containing 1.5 mol L^{-1} Na_2SO_4 spiked with 150 μg L^{-1} of each analyte without pH adjustment; vortex time in adsorption step, 5 min; desorption solvent (volume), ACN (1.0 mL); vortex time in desorption step, 5 min; and centrifugation speed and time, 6000 rpm and 5 min, respectively. DLLME procedure: aqueous phase, 5 mL deionized water without pH adjustment and salt addition; extraction solvent (volume), 1,2-DBE (28 μL); centrifugation rate, 6000 rpm; and centrifugation time, 5 min. The error bars show the minimum and maximum of three repeated determinations.

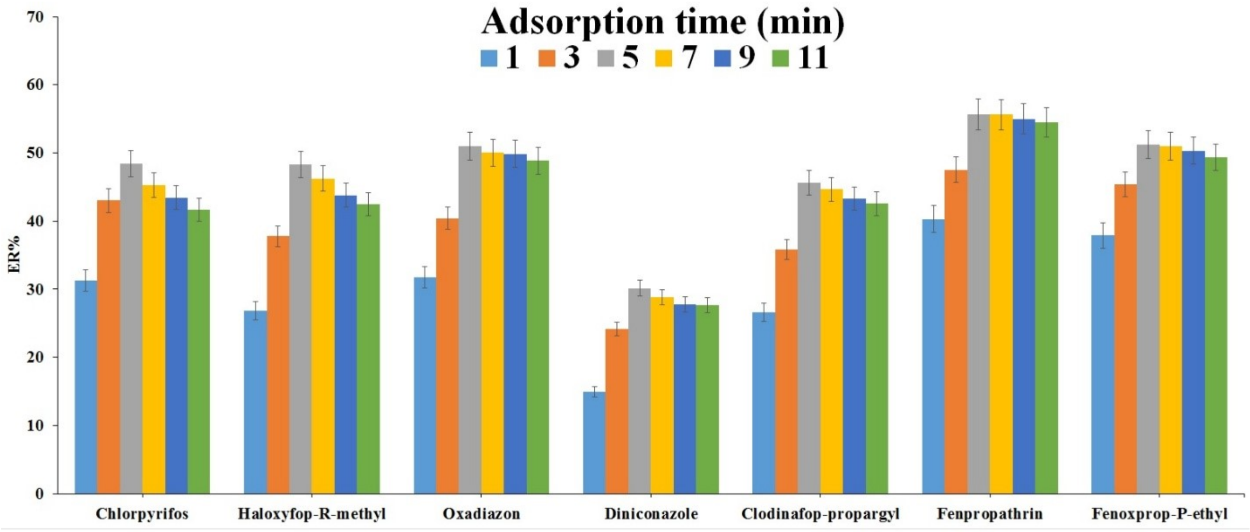

Fig. S2. Study of adsorption time.

Extraction conditions: are the same as those used in Fig. S1, without pH adjustment.

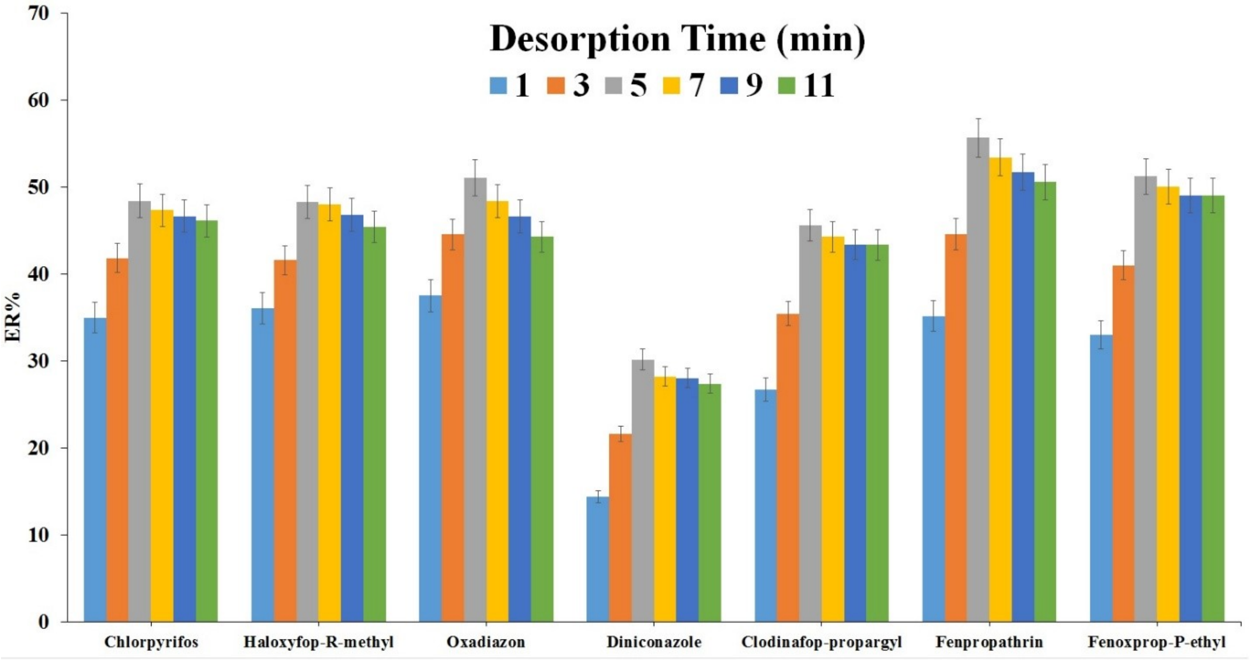

Fig. S3. Study of desorption time.

Extraction conditions: are the same as those used in Fig. S2, except 5 min was used as the adsorption time.

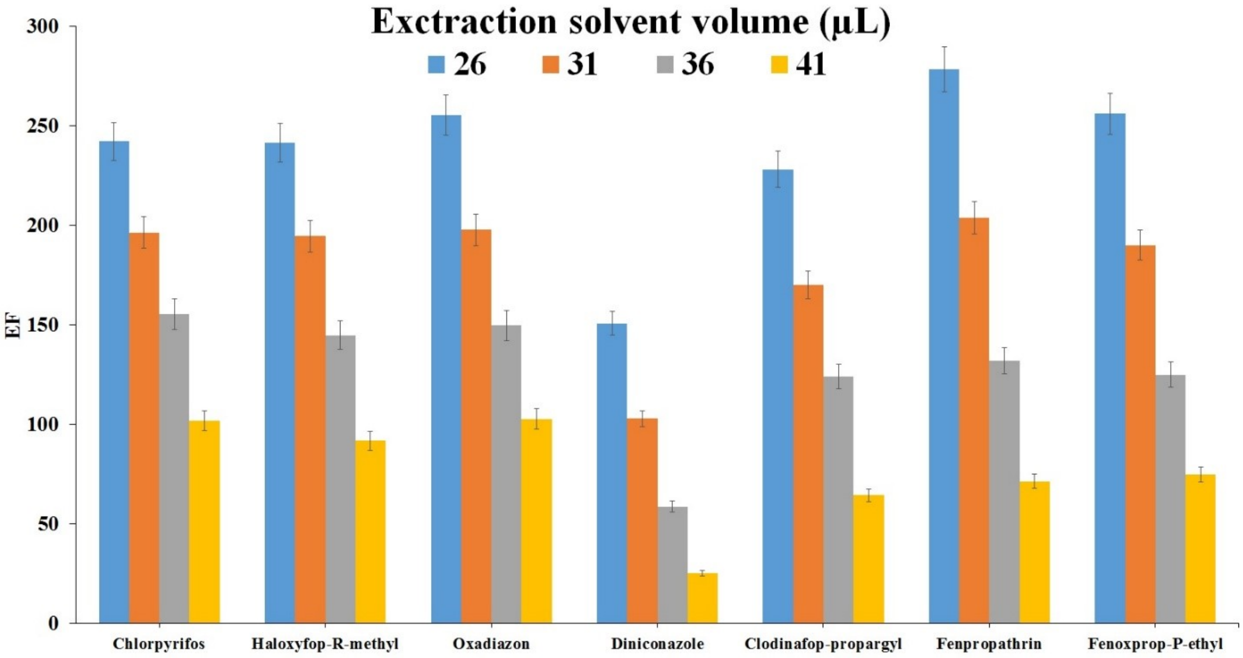

Fig. S4. Study of extraction solvent volume.

Extraction conditions: are the same as those used in Fig. S3, except 1,2-DBE and 5 min were used as the extraction solvent and desorption time, respectively.

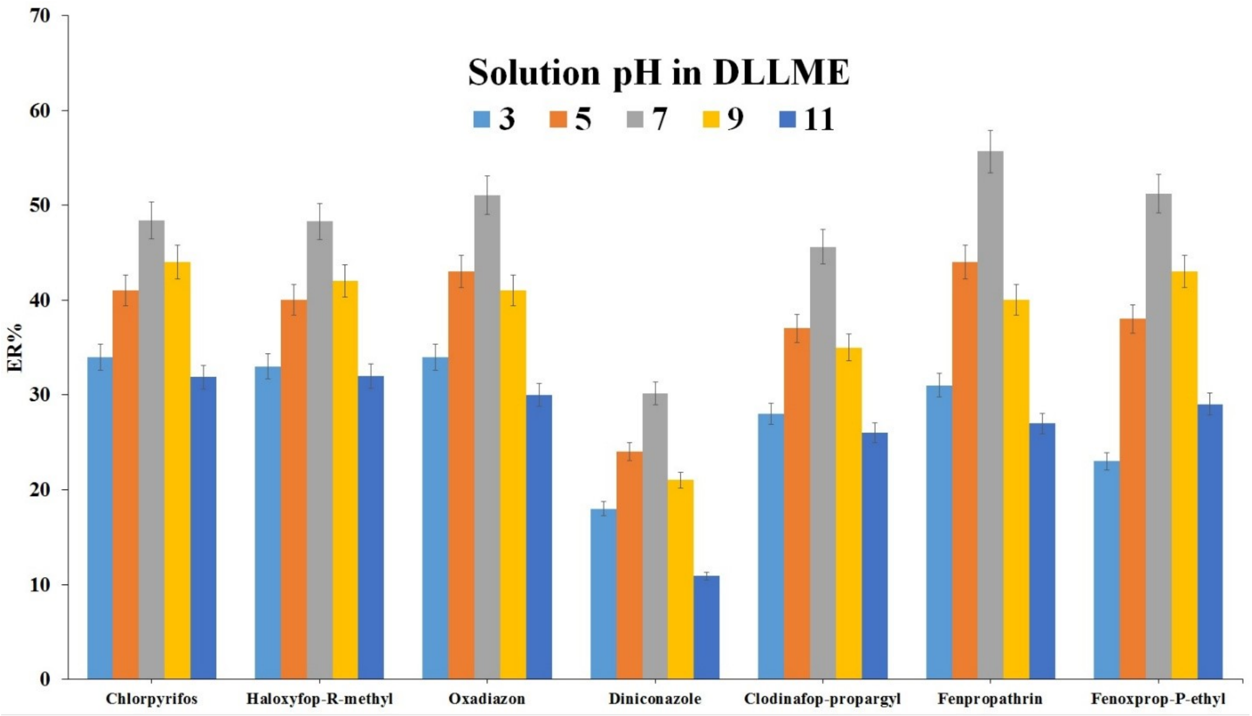

Fig. S5. Study of pH in DLLME.

Extraction conditions: are the same as those used in Fig. S4, except 26 μL 1,2-DBE was used as the extraction solvent.

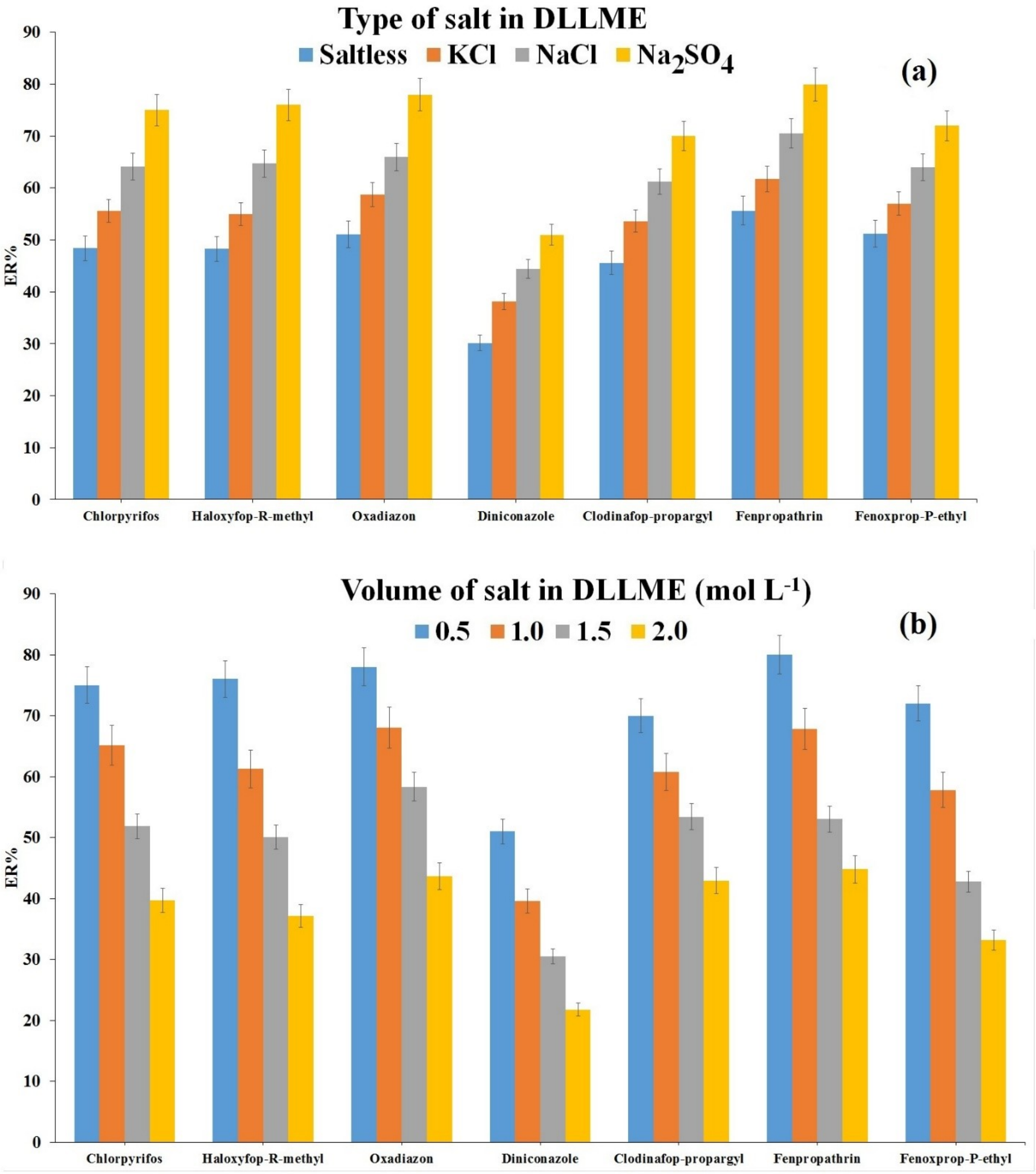

Fig. S6. impact of ionic strength in DLLME

Extraction conditions: are the same as those used in Fig. S5, except pH adjustment was not done in DLLME.
